# Invisible illness: The consequences of limited health insurance in Africa

**DOI:** 10.1002/hsr2.1313

**Published:** 2023-06-01

**Authors:** Aderinto Nicholas, Olatunji Deji

**Affiliations:** ^1^ Department of Medicine and Surgery Ladoke Akintola University of Technology Ogbomosho Nigeria; ^2^ Department of Medicine and Surgery University of Ilorin Ilorin Nigeria

**Keywords:** Africa, consequences, health insurance, underlying causes

## Abstract

**Background:**

Limited health insurance coverage in Africa poses a significant challenge, impeding access to quality healthcare for millions of individuals.

**Methods:**

This paper synthesizes information from existing literature and research on the topic of limited health insurance coverage in Africa. The identified consequences and root causes are presented in a structured format.

**Results:**

The consequences of limited health insurance coverage in Africa include increased financial burden on households, decreased access to health services, and inadequate coverage for essential health services. These consequences contribute to reduced utilization of healthcare services and negative health outcomes, including the deterioration of existing health conditions and the development of new health problems. The financial burden is particularly significant due to high poverty rates in Africa, forcing households to pay for healthcare services out of pocket and leading to considerable financial strain and even financial ruin. Additionally, limited health insurance coverage restricts access to necessary health services, resulting in delayed treatment, missed diagnoses, and poor health outcomes.

**Conclusion:**

The root causes of limited health insurance coverage in Africa are multifaceted and include factors such as poverty, lack of government support, and limited private‐sector involvement. These systemic issues contribute to the persistence of inadequate health insurance coverage and hinder efforts to improve access to quality healthcare for African populations.

## 1 INTRODUCTION

Limited health insurance coverage is a significant barrier to accessing quality healthcare services in Africa, despite the fundamental right to healthcare being recognized as a priority by many African nations.^1^ According to the World Health Organization (WHO), only about less than half of the African population has access to health insurance, with out‐of‐pocket payments (OOPs) accounting for about 30% of healthcare spending in Africa.^2^ This situation places an enormous financial burden on low‐income individuals and households.

The consequences of this limited access to health insurance in Africa are significant, with individuals without coverage facing obstacles in accessing quality healthcare services, leading to untreated conditions, delayed diagnoses, and poorer health outcomes.^3^ Additionally, healthcare providers cannot provide quality care to those who need it without a sustainable financing mechanism. The limited access to health insurance also impacts the healthcare system, leading to overburdened health facilities, shortages of medical supplies and personnel, and a decline in the quality of care provided.^4^ In sub‐Saharan Africa (SSA), a significant number of countries, specifically 27 out of 48, are affected by direct OOPs for healthcare services that exceed 30%, which annually impoverish over 100 million people, primarily in low‐ and middle‐income countries.^5^

To address the issue of catastrophic health expenditures, it is crucial to develop solutions that improve access to quality healthcare services and financial protection for all. This review aims to examine the consequences of limited health insurance coverage in Africa, identify the factors contributing to limited health insurance coverage, and identify potential strategies for improving health insurance coverage and access to healthcare services. By understanding the consequences of limited health insurance coverage, interventions can be developed and implemented to improve access to quality healthcare services and financial protection.

## 2 FACTORS CONTRIBUTING TO LIMITED HEALTH INSURANCE IN AFRICA

In Africa, poverty and limited access to health insurance are major barriers to universal health coverage. According to the World Bank, over 50% of the population in this region lives in extreme poverty, making it difficult to afford even basic healthcare services.^6^ Moreover, studies have shown that low‐income households are less likely to have health insurance coverage than higher‐income households, with cost being a significant barrier.^7^, ^8^ This situation seriously affects the health and well‐being of African individuals and communities. Without access to adequate healthcare services, preventable illnesses, and chronic conditions can go untreated, leading to unnecessary suffering and premature death.^4^ Moreover, out‐of‐pocket healthcare payments can drive people further into poverty, perpetuating a vicious cycle of poor health and economic insecurity^9^ (see Table 1). Addressing these challenges requires a comprehensive approach that tackles poverty and its underlying determinants.

**Table 1:** Comparison of health insurance models in Africa.

Health insurance model	Characteristics	Target population	Funding	Cost‐sharing	Effectiveness	Examples
Social health insurance	Mandated coverage for formal sector workers and their dependents, voluntary for informal sector	Formal sector workers and their dependents, some coverage for poor and vulnerable populations	Payroll taxes, government subsidies	Premiums, co‐payments, deductibles	High financial protection, limited coverage of informal sector	Ghana NHIS, Rwanda RSSB
Community‐based health insurance	Voluntary, membership‐based insurance schemes	Rural and informal sector populations	Membership fees, donor funding	Low premiums, cost‐sharing for some services	Limited financial protection, low coverage of formal sector	Mutuelles in Rwanda, Community Health Fund in Tanzania
Private health insurance	Voluntary participation, risk‐pooling	Formal sector employees and high‐income individuals	Premiums paid by individuals and employers	High administrative costs, potential for exclusion of low‐income individuals	Improved access to healthcare services, financial protection (for those who can afford it)	AAR Health Services (Kenya), Liberty Health (South Africa)

Limited government infrastructure is another significant factor contributing to the limited health insurance coverage in Africa. Without proper infrastructure, governments often cannot establish or effectively run health insurance programs, leading to limited coverage.^10^ In contrast, high‐income countries tend to have well‐developed government infrastructures, including robust healthcare systems and social safety nets. For instance, in the United States, the government sponsors various health insurance programs, including Medicare and Medicaid, which provide coverage to vulnerable populations.^11^ The lack of government infrastructure in Africa is evident in the limited number of health insurance programs available to the population.^12^ This lack of coverage is particularly acute in rural areas, where healthcare facilities and services are scarce. Without government intervention to improve healthcare infrastructure and establish effective health insurance programs, the situation will unlikely improve soon.

Furthermore, a limited understanding of health insurance contributes to the limited health insurance coverage in Africa. Many individuals in Africa have limited knowledge and understanding of health insurance, making it difficult for them to make informed decisions on whether or not to enroll in a plan.^13^ The limited comprehension of the various types of health insurance available also restricts access to adequate coverage that meets individual needs. In many cases, individuals are not aware of the benefits of health insurance or how to enroll in a plan. This lack of knowledge can lead to individuals choosing not to enroll in a plan or one that does not provide adequate coverage for their needs. In contrast, in high‐income countries such as the United States, health insurance is a fundamental part of the healthcare system.^14^ This high level of coverage results from various policies and initiatives aimed at increasing access to healthcare, including the Affordable Care Act (ACA), which expanded access to health insurance for millions of Americans.

Inadequate regulatory control is a significant factor contributing to the limited health insurance coverage in Africa. In the African insurance sector, proper regulatory control is lacking, creating a higher perception of risk among insurance companies, especially in the health insurance sector, where risks are heightened due to the unpredictable nature of health outcomes.^15^ The absence of adequate regulation increases the probability of insurance companies engaging in financial mismanagement, fraud, and other unethical practices that can undermine the sector's credibility and diminish consumer trust. The inadequate regulatory oversight also limits the ability of governments to monitor and enforce health insurance policies effectively, creating an environment where insurance companies are less incentivized to provide adequate coverage, as there are few consequences for noncompliance.^16^ The absence of consumer protection measures exposes vulnerable individuals to exploitation by insurance companies. In contrast to high‐income countries, where the insurance regulatory environment is well‐established, the regulatory environment in Africa's insurance sector is less developed, leading to lower levels of trust in the sector. According to the World Bank, only 26% of African countries have a strong regulatory environment for insurance, compared with 81% of high‐income countries.^17^ This indicates the need for African governments to establish robust regulatory frameworks that provide adequate oversight and consumer protection measures to enhance access to health insurance coverage in the region.

Furthermore, misallocating resources, theft of funds, and other unethical practices have contributed to inadequate and scarce healthcare facilities in Africa. As a result, the quality of care is often poor, with patients lacking access to basic medicines and medical supplies. The World Bank estimates that corruption in the health sector can increase the cost of healthcare by up to 40%.^18^ African governments must address corruption in the healthcare sector to improve the quality of care and increase the availability of health insurance programs for the population.

## 3 CONSEQUENCES OF LIMITED HEALTH INSURANCE COVERAGE IN AFRICA

Access to quality healthcare is a persistent challenge for many African individuals, with limited health insurance coverage as a significant barrier to healthcare access. The region suffers from a shortage of healthcare infrastructure, inadequate financing, and a shortage of healthcare workers, as highlighted by the WHO.^19^ Research indicates that limited health insurance coverage exacerbates healthcare inequalities.^20^ A study published in the *International Journal for Equity in Health* found that individuals with health insurance in Ghana had significantly better access to healthcare services and received better quality care than those without insurance.^21^ Additionally, out‐of‐pocket expenditures for healthcare in SSA are high.^5^ This financial burden can deter individuals from seeking care when they need it. Furthermore, the negative impact of limited health insurance coverage extends to healthcare providers. Providers without insurance struggle to cover their costs and sustain their practices, leading to a shortage of healthcare providers in certain regions and a decline in the overall quality of healthcare services.

Inadequate health insurance coverage has significant implications for healthcare utilization, as individuals may delay or forego seeking medical care due to the cost of treatment. This results in a lack of preventive care, including immunizations, a significant concern in Africa. According to the WHO, Africa accounts for 93% of the world's malaria cases and 71% of HIV/AIDS cases, highlighting the urgent need for preventive care measures.^22^ The lack of access to preventive care worsens health outcomes, particularly in vulnerable populations such as children and those with chronic conditions. For instance, malaria remains a leading cause of morbidity and mortality in Africa, accounting for over 400,000 deaths annually.^22^ Similarly, HIV/AIDS continues to pose a significant health threat, with over 25 million Africans living with the virus.^22^ Furthermore, inadequate insurance coverage can result in inadequate treatment due to a lack of resources, including medication, equipment, and personnel. The shortage of healthcare personnel also affects the ability of medical professionals to provide specialized care to patients, particularly in underserved regions. This exacerbates the problem of poor health outcomes, as patients with chronic conditions such as diabetes or hypertension are particularly vulnerable to inadequate treatment. The consequences of inadequate insurance coverage on healthcare utilization, treatment, and outcomes are particularly concern given the significant burden of disease in Africa. Therefore, policymakers and stakeholders must prioritize interventions that expand access to affordable and comprehensive health insurance, increase healthcare services in underserved regions, and leverage innovative financing mechanisms to achieve universal health coverage.

Similarly, inadequate health insurance coverage can significantly impact household financial burden, particularly in Africa, where OOPs for healthcare services are prevalent. The World Bank reports that in SSA, OOPs account for 35% of total health expenditures, compared with 13% in high‐income countries.^2^ The high healthcare costs associated with limited health insurance coverage result in financial hardship for households and reduced access to essential goods and services, such as food and education. The financial burden of limited health insurance coverage particularly concerns vulnerable populations, such as low‐income households and those with chronic conditions, who are more likely to experience high healthcare costs. According to a study published in Health Policy, households in Ghana with a member with a chronic condition spend, on average, 14% of their income on healthcare costs.^21^ Moreover, high healthcare costs can lead to households forgoing essential goods and services, resulting in increased poverty levels and reduced overall well‐being.

## 4 ADDRESSING THE CHALLENGES OF LIMITED HEALTH INSURANCE IN AFRICA

Access to health insurance remains a significant challenge for many African individuals, with limited coverage exacerbating existing healthcare inequalities. Various strategies, including social health insurance programs, community‐based health insurance schemes, microinsurance, technology‐based solutions, and regulatory reforms, have driven efforts to expand health insurance coverage in the region.

Several countries in Africa have implemented social health insurance programs, such as Ghana, Rwanda, and Senegal, which aim to provide affordable health insurance coverage to low‐income households, particularly those in rural and underserved areas. These programs have been successful in expanding access to health insurance coverage and improving healthcare utilization and outcomes.^23^ Community‐based health insurance programs have also been implemented in Kenya, Uganda, and Tanzania to provide affordable health insurance coverage to individuals and families in rural and underserved areas. These programs are designed and managed by communities or community‐based organizations and have been successful in expanding access to health insurance coverage and improving healthcare utilization and outcomes.^24^ Microinsurance is also emerging as a promising strategy for improving access to health insurance in Africa, with various organizations, including microfinance institutions, NGOs, and insurance companies, implementing microinsurance products that are affordable and accessible to low‐income households. Technology‐based solutions, such as mobile technology, are being used to facilitate the enrollment and payment of health insurance premiums. For instance, the M‐TIBA platform in Kenya enables users to save and access funds for healthcare services, facilitates the payment of health insurance premiums, and provides a digital record of healthcare transactions.^25^

While progress has been made in expanding access to health insurance through various strategies, future efforts should focus on several key areas. There is a need to strengthen healthcare infrastructure and systems, particularly in rural and underserved areas. This includes improving the availability and quality of healthcare facilities, equipment, and personnel, as well as strengthening health information systems to support the implementation and monitoring of health insurance programs. In addition, efforts should be made to improve the affordability and accessibility of health insurance products for low‐income households. This can be achieved through the development of innovative and flexible health insurance products, as well as the provision of subsidies and financial assistance to low‐income households to enable them to access health insurance.

Furthermore, there is a need to increase public awareness and education about the benefits of health insurance and the available options (see Table 2). This can be achieved through targeted public education campaigns and the involvement of community leaders and health workers in promoting health insurance enrollment and utilization. There is a need for increased collaboration and partnership between governments, private sector stakeholders, and civil society organizations to support the expansion and sustainability of health insurance programs in Africa. This includes the development of public‐private partnerships to leverage resources and expertise, as well as the engagement of civil society organizations to promote community participation and ownership of health insurance programs.

**Table 2:** Key factors and policy recommendations for limited health insurance coverage Africa.

Factor	Example	Policy recommendation
High poverty rates	In many African countries, a significant portion of the population lives in poverty and cannot afford health insurance premiums.	Develop targeted subsidies or vouchers to support low‐income individuals and families in accessing health insurance. Increase funding for social health insurance programs to expand coverage to vulnerable populations.
Lack of healthcare infrastructure	Many African countries have limited healthcare infrastructure, which makes it difficult to implement and support health insurance programs.	Increase investment in healthcare infrastructure to support the development and expansion of health insurance programs. This could include funding for the construction of healthcare facilities and the training of healthcare workers. Develop public‐private partnerships to leverage private sector resources and expertise in building healthcare infrastructure.
Limited regulatory frameworks	Many African countries lack robust regulatory frameworks for health insurance, which can hinder the development and implementation of effective programs.	Provide technical assistance to national governments in developing and implementing regulatory frameworks for health insurance. This could involve supporting the development of legislation, regulations, and guidelines for health insurance, as well as providing training and capacity building for regulatory agencies.
Fragmented healthcare systems	In many African countries, healthcare systems are fragmented and decentralized, which can make it difficult to implement and manage health insurance programs.	Develop coordinated approaches to healthcare delivery that involve collaboration between different healthcare providers and stakeholders. This could involve the development of integrated health systems or the establishment of referral networks to facilitate access to specialized care.
Limited public awareness	Many individuals in Africa are not aware of the benefits of health insurance or how to access it.	Develop public education campaigns to raise awareness about the benefits of health insurance and how to enroll in programs. Use community‐based approaches to engage individuals and families in health insurance enrollment and utilization.
Political instability	Political instability and conflict can undermine the development and implementation of health insurance programs.	Work with national and international partners to address underlying political and security challenges that impact healthcare delivery. Provide technical and financial support to strengthen healthcare systems in conflict‐affected areas.

International aid and development agencies have the potential to contribute significantly toward addressing the limited health insurance coverage in Africa. Such agencies can offer technical assistance, funding, and other resources to support the development and implementation of health insurance programs in the region. One area of potential support is in developing regulatory frameworks for health insurance. International agencies can provide technical assistance to national governments in developing and implementing regulatory frameworks for health insurance. This could entail supporting the development of legislation, regulations, and guidelines for health insurance, as well as providing training and capacity building for regulatory agencies. Additionally, international agencies could provide funding and technical assistance to support the expansion of social health insurance and community‐based health insurance programs in Africa. This could involve the establishment of risk pools, subsidies for premium payments, and technical assistance to improve the efficiency and effectiveness of these programs. Moreover, international aid and development agencies can support developing and implementing technology‐based solutions to enhance access to health insurance. This could entail funding for the development of mobile health platforms, integrating these platforms with health insurance programs, and providing training and capacity building for healthcare workers and consumers in using these platforms.

## 5 CONCLUSION

The limited health insurance coverage in Africa poses significant challenges to healthcare services in the region. However, promising strategies can be pursued to expand access to health insurance, including the development of regulatory frameworks, the expansion of community‐based and social health insurance programs, the use of technology‐based solutions, and efforts to address broader structural and systemic issues in the healthcare system. International aid and development agencies are important in supporting these efforts through technical assistance, funding, and other resources. By working collaboratively with national governments and other stakeholders, Africa can progress toward achieving universal health coverage and improving health outcomes in Africa.

## AUTHOR CONTRIBUTIONS

**Aderinto Nicholas**: Conceptualization; methodology; project administration; software; supervision; writing—original draft; writing—review and editing. **Olatunji Deji**: Data curation; methodology; project administration; software; validation; writing—original draft; writing—review and editing.

## CONFLICT OF INTEREST STATEMENT

The authors declare no conflict of interest.

## TRANSPARENCY STATEMENT

The lead author Aderinto Nicholas affirms that this manuscript is an honest, accurate, and transparent account of the study being reported; that no important aspects of the study have been omitted; and that any discrepancies from the study as planned (and, if relevant, registered) have been explained.

## Data Availability

Data sharing not applicable to this article as no data sets were generated or analyzed during the current study.

## References

[1] Oleribe OE , Momoh J , Uzochukwu BS , et al. Identifying key challenges facing healthcare systems in Africa and potential solutions. Int J Gen Med. 2019;12:395‐403. 10.2147/IJGM.S223882 31819592 PMC6844097

[2] Tessema ZT , Worku MG , Tesema GA , et al. Determinants of accessing healthcare in Sub‐Saharan Africa: a mixed‐effect analysis of recent Demographic and Health Surveys from 36 countries. BMJ Open. 2022;12(1):e054397. 10.1136/bmjopen-2021-054397 PMC8804632 35105635

[3] Azevedo MJ . The state of health system(s) in Africa: challenges and opportunities. Historical Perspectives on the State of Health and Health Systems in Africa, Volume II: The Modern Era, 2017:1‐73. 10.1007/978-3-319-32564-4_1

[4] Kruk ME , Gage AD , Arsenault C , et al. High‐quality health systems in the Sustainable Development Goals era: time for a revolution. Lancet Glob Health. 2018;6(11):e1196‐e1252. 10.1016/S2214-109X(18)30386-3 30196093 PMC7734391

[5] Ifeagwu SC , Yang JC , Parkes‐Ratanshi R , Brayne C . Health financing for universal health coverage in Sub‐Saharan Africa: a systematic review. Glob Health Res Policy. 2021;6:8. 10.1186/s41256-021-00190-7 33641673 PMC7916997

[6] Adeyeye SAO , Ashaolu TJ , Bolaji OT , Abegunde TA , Omoyajowo AO . Africa and the Nexus of poverty, malnutrition and diseases. Crit Rev Food Sci Nutr. 2023;63(5):641‐656. 10.1080/10408398.2021.1952160 34259104

[7] Allen EM , Call KT , Beebe TJ , McAlpine DD , Johnson PJ . Barriers to care and health care utilization among the publicly insured. Med Care. 2017;55(3):207‐214. 10.1097/MLR.0000000000000644 27579910 PMC5309146

[8] National Academies of Sciences, Engineering, and Medicine . Factors that affect health‐care utilization. Health‐Care Utilization as a Proxy in Disability Determination. National Academies Press; 2018:5‐25. 29782136

[9] Aregbeshola BS , Khan SM . Out‐of‐pocket payments, catastrophic health expenditure and poverty among households in Nigeria 2010. Int J Health Policy Manag. 2018;7(9):798‐806. 10.15171/ijhpm.2018.19 30316228 PMC6186489

[10] Onwujekwe O , Ezumah N , Mbachu C , et al. Exploring effectiveness of different health financing mechanisms in Nigeria; what needs to change and how can it happen? BMC Health Serv Res. 2019;19:661. 10.1186/s12913-019-4512-4 31519181 PMC6743191

[11] Alegría M , Cao Z , McGuire TG , et al. Health insurance coverage for vulnerable populations: contrasting Asian Americans and Latinos in the United States. INQUIRY J Health Car. 2006;43(3):231‐254. 10.5034/inquiryjrnl_43.3.231 PMC2712944 17176967

[12] Deaton AS , Tortora R . People in sub‐Saharan Africa rate their health and health care among the lowest in the world. Health Aff. 2015;34(3):519‐527. 10.1377/hlthaff.2014.0798 PMC5674528 25715657

[13] Akokuwebe ME , Idemudia ES . A comparative cross‐sectional study of the prevalence and determinants of health insurance coverage in Nigeria and South Africa: a multi‐country analysis of demographic health surveys. Int J Environ Res Public Health. 2022;19(3):1766. 10.3390/ijerph19031766 35162789 PMC8835528

[14] Papanicolas I , Woskie LR , Jha AK . Health care spending in the United States and other high‐income countries. JAMA. 2018;319(10):1024‐1039. 10.1001/jama.2018.1150 29536101

[15] Institute of Medicine (US) Committee on the Consequences of Uninsurance . Effects of health insurance on health. Care Without Coverage: Too Little, Too Late. National Academies Press; 2002:20‐67. 25057604

[16] van den Heever AM . The role of insurance in the achievement of universal coverage within a developing country context: South Africa as a case study. BMC Public Health. 2012;12(suppl 1):S5. 10.1186/1471-2458-12-S1-S5 22992410 PMC3381693

[17] World Bank . Universal health coverage in Africa: a framework for action. 2016. https://www.worldbank.org/en/topic/universalhealthcoverage/publication/universal-health-coverage-in-africa-a-framework-for-action

[18] World Bank . Universal health coverage. 2021. https://www.worldbank.org/en/topic/universal.health.coverage

[19] World Health Organization Regional Office for Africa . Chronic staff shortfalls stifle Africa's health systems ‐ WHO study. August 30, 2018. https://www.afro.who.int/news/chronic-staff-shortfalls-stifle-africas-health-systems-who-study

[20] Fiestas Navarrete L , Ghislandi S , Stuckler D , Tediosi F . Inequalities in the benefits of national health insurance on financial protection from out‐of‐pocket payments and access to health services: cross‐sectional evidence from Ghana. Health Policy Plan. 2019;34(9):694‐705. 10.1093/heapol/czz093 31539034 PMC6880330

[21] Dake FAA . Examining equity in health insurance coverage: an analysis of Ghana's National Health Insurance Scheme. Int J Equity Health. 2018;17:85. 10.1186/s12939-018-0793-1 29914497 PMC6006705

[22] World Health Organization . *World Malaria Report 2019*. December 4, 2019. https://www.who.int/news-room/feature-stories/detail/world-malaria-report-2019

[23] Ly MS , Bassoum O , Faye A . Universal health insurance in Africa: a narrative review of the literature on institutional models. BMJ Glob Health. 2022;7(4):e008219. 10.1136/bmjgh-2021-008219 PMC9052052 35483710

[24] Ogben C , Ilesanmi O . Community based health insurance scheme: preferences of rural dwellers of the federal capital territory Abuja, Nigeria. J Public Health Afr. 2018;9(1):540. 10.4081/jphia.2018.540 30079158 PMC6057720

[25] Huisman L , van Duijn SM , Silva N , et al. A digital mobile health platform increasing efficiency and transparency towards universal health coverage in low‐ and middle‐income countries. Digit Health. 2022;8:205520762210922. 10.1177/20552076221092213 PMC9005819 35433018

